# Biodiversity is overlooked in the diets of different social groups in Brazil

**DOI:** 10.1038/s41598-023-34543-8

**Published:** 2023-05-09

**Authors:** Sávio Marcelino Gomes, Viviany Moura Chaves, Aline Martins de Carvalho, Elenilma Barros da Silva, Elias Jacob de Menezes Neto, Gabriela de Farias Moura, Leonardo da Silva Chaves, Rômulo Romeu Nóbrega Alves, Ulysses Paulino de Albuquerque, Fillipe de Oliveira Pereira, Michelle Cristine Medeiros Jacob

**Affiliations:** 1 Department of Nutrition, Federal University of Paraiba, Street Tabelião Stanislau Eloy, s/n, Castelo Branco, João Pessoa, PB 58050-585 Brazil; 2 Multicampi School of Medical Sciences, Federal University of Rio Grande do Norte, Caicó, RN Brazil; 3 Department of Nutrition, School of Public Health, University of São Paulo, Av. Dr Arnaldo, 715, São Paulo, SP 01246-904 Brazil; 4 Restaurante Universitário–Federal University of Para, Rua Algusto Corrêa, 01, Belém, Pará 66075-110 Brazil; 5 Metropole Digital Institute, Federal University of Rio Grande do Norte, Natal, RN Brazil; 6 LabNutrir, Nutrition Department, Federal University of Rio Grande do Norte, Av. Senador Salgado Filho, s/n, Lagoa Nova, Natal, RN 59078-970 Brazil; 7 Escola de Educação e Humanidades, Universidade Católica de Pernambuco, Rua do Príncipe, n. 526, Boa Vista, Recife, Pernambuco Brasil; 8 Museu de Arqueologia e Ciências Naturais da Universidade Católica de Pernambuco, Rua Oliveira Lima, 824, Boa Vista, Recife, Pernambuco Brasil; 9 Departamento de Biologia, Universidade Estadual da Paraíba, Campina Grande, Paraíba 58019-753 Brazil; 10 Laboratório de Ecologia e Evolução de Sistemas Socioecológicos, Departamento de Botânica, Universidade Federal de Pernambuco, Recife, Pernambuco 50670-901 Brazil; 11 FUNGI Research Group, Academic Unit of Health, Education and Health Center, Federal University of Campina Grande, Sítio Olho D’agua da Bica, s/n, Cuité, PB 58175-000 Brazil

**Keywords:** Biodiversity, Nutrition, Public health

## Abstract

Food biodiversity is essential for improving nutrition and reducing hunger in populations worldwide. However, in middle and low-income countries, the biodiversity of food production does not necessarily represent food consumption patterns by population. We used Brazil, one of the world's megabiodiverse countries, as a case study to investigate the following questions: what is the prevalence of consumption of biodiverse foods in Brazil, and what are the socioeconomic factors that influence their consumption throughout the country? We used data from a Brazilian representative national dietary survey to estimate the frequency of food consumption of unconventional food plants, edible mushrooms, and wild meat, in according to socioeconomic variables. Thus, we investigated the socioeconomic predictors of Unconventional Food Plants consumption using methods of Machine Learning (ML) and multiple zero-inflated Poisson (ZIP) regression. We showed that biodiverse food consumption in Brazil is low, just related by 1.3% of the population, varying in according to area, ethnicity, age, food insecurity, sex, and educational level. Our findings of low utilization of biodiversity suggest an important mismatch between the rich biodiversity of the country and its representation in the human diet.

## Introduction

Studies analyzing biodiversity in food systems have consistently identified a discernible trend towards uniformity. This trend is exemplified by a meta-analysis study that analyzed food consumption data from 59 lower-income countries. The study found a yearly 4% decline in dietary diversity among children aged 1 to 5^1^. Similarly, the most comprehensive analysis of global food supply diversity conducted over the past 50 years demonstrates that national food supplies around the world have become increasingly similar in composition^2^. The fact that 50 agricultural commodities, such as wheat, corn, and rice, contribute to 90% of the world's calories, protein, and fat further underscores this trend of homogenization.

All of these studies emphasize the detrimental effect of decreased access to biodiverse foods, which include a range of wild and/or underutilized plants, fungi, algae, and wild animals (in specific contexts)^3^, on nutritional diversity. Biodiverse foods play a crucial role in protecting food security by supplementing staple crops and providing essential micronutrients. For instance, a study conducted in rural areas of Tanzania revealed that although wild foods contribute only 2% of total energy in the diet, they contribute large percentages of vitamin A (RAE) (31%), vitamin C (20%), and iron (19.19%)^4^. Another study in central Brazilian Amazonia indicates that among rural children who are most vulnerable to poverty, wild meat consumption is associated with higher hemoglobin concentration^5^. Recent studies also demonstrate that mushrooms and algae are promising sustainable and nutritious food sources, with some offering vitamins such as B12 previously thought to be exclusive to animal-origin foods^6,7^. Therefore, consuming biodiverse foods can have a positive impact on achieving both Sustainable Development Goals (SDG) 2 (zero hunger and improved nutrition) and SDG15 (life on land and biodiversity conservation). This is because agricultural diversity and wild biodiversity have the potential to make food systems more nutrition-sensitive and foster food security^8^.

Despite the potential for countries with high biodiversity to increase dietary quality, recent studies have shown that a biodiverse-rich environment does not necessarily result in a diverse diet. For instance, a study conducted in the Democratic Republic of Congo revealed that wild edible plants were insufficiently consumed to increase the dietary adequacy^9^. In Indonesia, a megadiverse country, a study that analyzed three waves of a panel data set from the Indonesian Family Life Survey with a balanced sample of 2785 rural households covering the period between 2000 and 2015 found a similar decline in dietary diversity over time^10^. For the authors, the lack of access to wild foods (meats, fruits, and leafy vegetables) produced a negative impact on dietary diversity.

Brazil is a megabiodiverse country^11^, home to more than 50,000 native species of plants, fungi, and algae, and over 100,000 species of animals. Despite this remarkable biodiversity, there is limited knowledge about the extent to which it contributes to the average Brazilian diet. Moreover, the literature does not provide a clear consensus on the factors that influence the consumption of biodiverse foods. While some studies suggest that overall dietary diversity is declining in lower-income countries regardless of individual wealth or location (urban/rural)^1^, other research indicates that increasing income can lead to more diverse diets, particularly in rural areas^12,13^. Regarding wild foods, analysis of the Brazilian case suggests an inverse relationship between income and consumption of wild edible plants, particularly in rural contexts. For instance, Cruz et al.^14^ found that in communities in the Brazilian semi-arid region, families with lower incomes possess more knowledge of and consume more wild edible plants. However, this finding is not universally agreed upon, as shown by the conflicting results of Nascimento et al.^15^. To fill these gaps in knowledge, our study aimed to answer two main questions: what is the prevalence of consumption of biodiverse foods in Brazil, and what are the socioeconomic factors that influence their consumption throughout the country? This study is, to the best of our knowledge, the first to measure the contribution of biodiverse foods, specifically plants, fungi, and meat, using a representative sample of the Brazilian population.

## Methods

### Data source

We used data from the official Brazilian National Dietary Survey of a sub-sample of respondents in the Household Budget Survey (NDS-HBS) conducted by the Brazilian Institute of Geography and Statistics (IBGE) between July 2017 and July 2018. These data are public and available by IBGE; no humans were directly involved in our study.

The 2017–2018 IBGE dietary survey sampling was defined by clusters, in two stages: in the first, census tracts were drawn; in the second, households were drawn within each extract. The final sample included 57,920 Brazilian households. The NDS-HBS assessed the food intake using 24-h food recalls, on two non-consecutive days, in a random subset of 34.7% of households, totaling 46,164 individuals aged 10 years or over. More information about the sampling process can be found in official publications of the IBGE^16^.

### Food groups and variables

#### Food groups

All subjects reported all foods and beverages consumed the day before both interviews, including information on ingredients, preparation, and quantities. We categorized food reported into three groups: UFP, edible mushrooms, and wild meat. We excluded edible algae from our analysis due to the lack of sufficient observations (n = 1) in the database.

#### UFP

Since there is no consensus list of UFP, we used the consensus of experts to classify the food plants reported by our sample. Initially, we selected all the plants consumed by the survey participants (219 plants) and sent them to six researchers with recognized scientific production on UFP from different Brazilian biomes. The invited researchers classified the 219 plants according to the following criteria: (1) limited use, either in geographical or cultural terms. (2) Potential to contribute to the food and nutritional security of human populations. (3) Potential to contribute to the sustainable use of biodiversity. We considered UFP those plants which more than 60% of the invited experts considered meeting criterion 1, and alternatively the criteria 2 or 3.

#### Mushroom

As there was no report of edible mushrooms by species in the dietary survey, we included the following reported foods: uncooked mushrooms, preserved mushrooms, and fungi risotto meals.

#### Wild meat

We considered wild meat or bushmeat as meat derived from any wild animals, especially non-aquatic vertebrates, harvested for subsistence or trade, excluding fish^17^. In this sense, we included in the bushmeat category wild animals used as a food resource, as well as preparations including meat from these animals as an ingredient.

We collected the vernacular name, regions of occurrence, and frequency of consumption for UFP and wild meat. We used this information to classify the UFP and the bushmeat by genus and species (if possible). Using vernacular names and the location of consumption provided by the dietary survey and the location of the species occurrence provided by the Flora do Brasil database (https://floradobrasil.jbrj.gov.br/), we produced taxonomic clues that are proxies of scientific names of the species consumed.

We considered the mean of consumption in 2 days for all food groups. We only included recipes that mentioned foods in one of the three groups, and we used the Table of Reference Measures for Food Consumed in Brazil^18^ to estimate the amounts consumed in grams or milliliters of each food or beverage.

#### Socioeconomic and demographic variables

We used the variables sex (male or female), age (years), states of Brazil (names), degree of urbanization of households (urban or rural), education (years of schooling), per capita income (USD), and household state of food insecurity. We used the variable 'ethnicity' in two ways: (1) for descriptive analysis in its original form, using the categories of the national survey, including white, black, asian, multiethnic, or indigenous; and (2) for the models, we recategorized it into 'white' and 'non-white'. The latter category (i.e., non-white) included black, asian, multiethnic, and indigenous groups to create a group that potentially combines similar experiences of oppression based on race, as described in Wood et al.^19^ and Skidmore^20^. The IBGE survey measured food insecurity according to the reduced eight item version of the Brazilian Food Insecurity Scale (EBIA), the official Brazilian tool to determine food insecurity levels in the population. We classified degrees of food insecurity based on the final scale score, with the following cutoff points: Food security (0), mild food insecurity (1–3), moderate food insecurity (4–5), and severe food insecurity (6–8)^21^.

### Data analysis

#### Descriptive analysis

We performed descriptive analysis to describe the food groups and socioeconomic and demographic variables, using relative frequencies, means, and 95% confidence intervals. We accounted for the sample weights in order to accurately represent the study population according to the sample design of the research. We conducted these analyses using the R language through the RStudio interface version 2022.12.0-353 with the assistance of the ‘Survey’ package^22^.

#### Identifying socioeconomic predictors of unconventional food plants consumption

In addition to analyzing the overall consumption of biodiverse foods, we specifically analyzed the data on UFP to identify the socioeconomic predictors of their consumption. Unfortunately, we were unable to perform a similar analysis on wild meat and edible mushrooms due to the lack of observations related to the consumption of these food resources (consumption frequency < 1% of the total sample).

To choose the classifier with the greatest ability to model the phenomenon, we tested various machine learning architectures. To conduct our evaluations, we first had to balance the original dataset. The original data showed a significant imbalance between our target variables, with the negative class comprising 45,546 instances and the positive class only having 618 observations. To fix this, we applied a random undersampling method to the majority class data, resulting in a sample of 927 data points from that class. The new dataset now had a more balanced distribution of instances between the positive and negative classes, with one-third belonging to the positive class and two-thirds belonging to the negative class. We deliberately maintained a slight imbalance in the data to better capture the complexities of the problem under analysis.

We normalized the independent variables so that the values ranged from -1 to 1. Data normalization is a common requirement for many machine learning estimators. To normalize, the standard score of the sample was calculated as follows: $z= \frac{(x-u)}{s}$, where $x$ represents the independent variables, $u$ is the mean of the training samples, and $s$ is the standard deviation of the training samples. We divided the entire dataset into ten groups of similar size using a stratified K-fold strategy, where K equals 10. This meant that the data was divided into ten groups, with each group having a similar composition of the dependent variable. To obtain the predicted values for each group, we trained the remaining nine groups and selected the classifier that showed the best Matthews Correlation Coefficient (MCC). The MCC is a measure of the quality of binary classifications, and it evaluates the differences between expected and predicted values. It is especially useful for imbalanced datasets, as it considers all elements from the confusion matrix.

After selecting the best classifier, we trained a new instance of the model using the entire dataset for SHAP (SHapley Additive exPlanations) value analysis. SHAP is a method based on cooperative game theory that increases the transparency and interpretability of machine learning models. The procedure we adopted aimed at (1) selecting the best classifier to approximate the phenomenon and (2) evaluating the importance of each independent variable for the result of the model.

We determined that the best combination for approximating the phenomenon was a Stacked model consisting of a Logistic Regression and a Catboost classifier. This architecture effectively blends linear and non-linear modeling approaches, and was selected as the best option due to its superior performance as indicated by the Matthews Correlation Coefficient (MCC), as shown in Supplementary Table 1. All the analyses were carried out using Python with the support of the following libraries: scikit-learn^23^, Pandas^24^, CatBoost^25^, and SHAP^26^.

Then, we used the multiple zero-inflated Poisson (ZIP) regression model to verify the association among UFP consumption and predict variables. This is a mixture model used to analyze skewed distribution with large proportion of zeros, and it estimates the distribution of the outcome by combining two distributions: a logistic regression model for the zero portion of the model and a Poisson regression for the count portion of the model^27^. The results of the ZIP model are presented as (log)β regression coefficients, their standard errors, and their p values, all related to the count portion of the model.

To use the ZIP model, we transformed the continuous variable of UFP consumption (g/day) into a count variable (number of UFP servings adjusted by kilocalories). We defined a serving size of UFP as the intake of 30 g/1000 kcal of UFP, based on the average intake of the population. We included the same covariates as in the SHAP model in our ZIP model: area, ethnicity, age, food insecurity, per capita income, sex, and educational level. We also accounted for the complexity of the sample to represent the entire population. We have included this solution based on traditional statistical methods in our analysis to make the article more accessible to a broader group of researchers who may not be familiar with machine learning techniques.

## Results

### Biodiverse foods in the Brazilian diet

**Figure 1:**
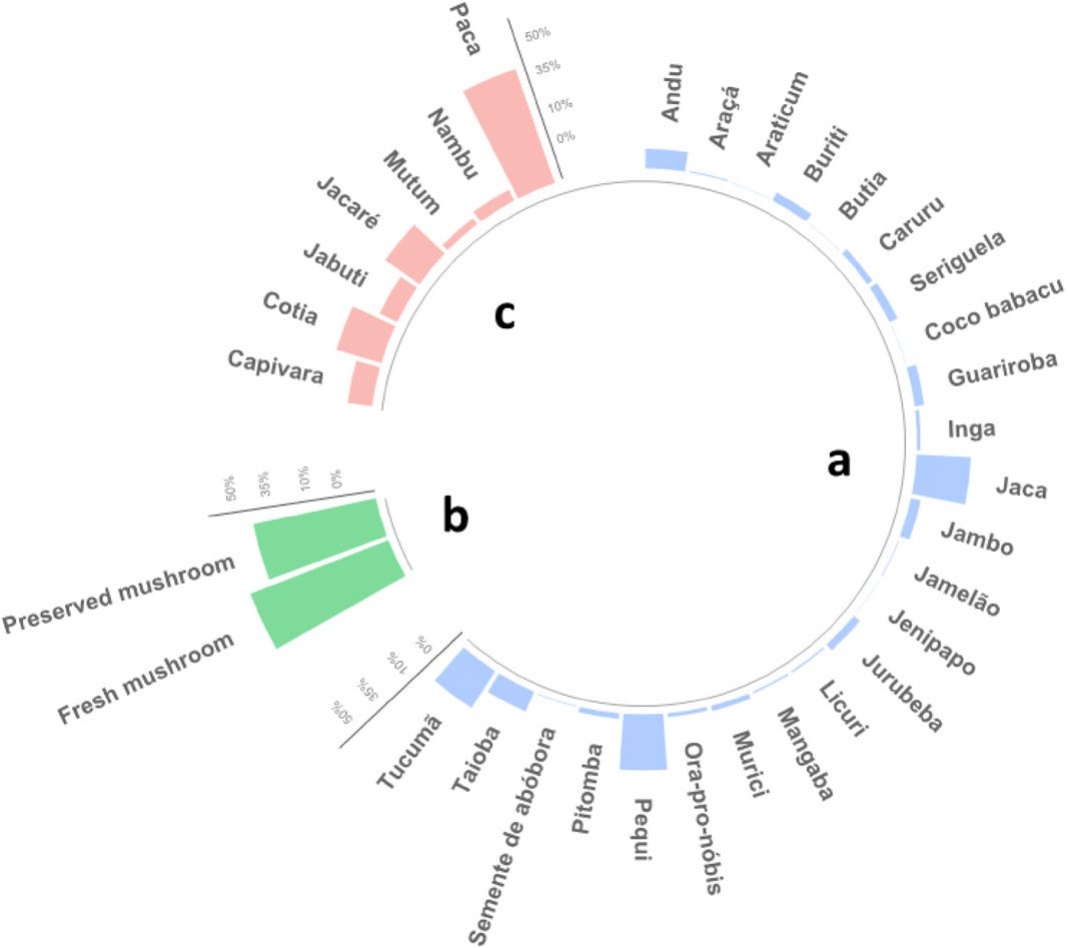
Frequency of biodiverse foods types for subsamples who related consumption of UFP, edible mushrooms, and wild meat, according to the National Dietary Survey—Household Budget Survey, 2017–2018. Group a: unconventional food plants (n = 618); Group b: mushrooms (n = 24); Group c: wild meat (n = 86).

Based on the evaluation and classification by six experts, of the 219 food plants reported in the NDS-HBS 2017–2018, the UFP represented 10% of all plants consumed by the Brazilian population. Of these plants, 24 were classified as UFP (Fig. 1). The most consumed UFP were andu (*Cajanus cajan* (L.) Mill), jaca (*Artocarpus heterophyllus* Lam.), pequi (*Caryocar* spp. L.), taioba (*Xanthosoma taioba* E.G.Gonç. or *Colocasia esculenta* (L.) Schott), and tucumã (*Astrocaryum aculeatum* G. Mey.). The highest frequency of consumption of these foods occurred in the states of Bahia (andu and jaca), Goiás (pequi), Minas Gerais (taioba), and Amazonas (tucumã), see Supplementary Table 2 and Supplementary Table 3.

We identified the consumption of seven different species of wild animals in the report. The paca (*Cuniculus paca* L.) and the cotia (*Dasyprocta* spp.) were the most consumed wild animals. Consumption was mainly reported in the state of Acre (North of Brazil) for both species. Mushrooms were mentioned only in a generic way, without any clues that would allow us to infer their taxonomies.

### Sociodemographic characteristics of people who consumed and not consume biodiverse foods

**Figure 2:**
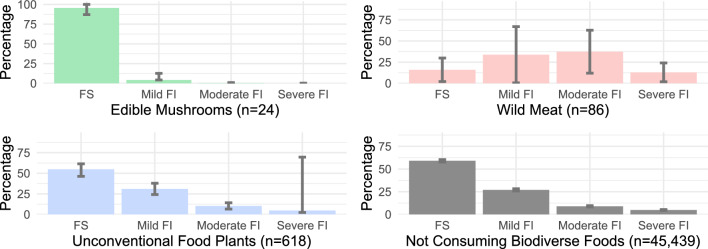
Levels of food security in the households of mushroom consumers, wild meat consumers, and unconventional food plant consumers, compared to a reference group of people who did not report consuming the biodiverse foods analyzed in this study, with a 95% confidence interval.

We estimated that 1.34% of the population consumed UFP, mushrooms, or wild meat (Table 1). The population who consumed UFP was composed of a higher percentage of women (61.24%) and a low percentage of white people (29.29%), with greater representation in the North and Northeast regions (15.10% and 40.04%, respectively). About 50% of our sample experienced food security (Fig. 2). The average estimates for age, years of schooling, and income did not show differences when compared to the averages of the Brazilian population, in observation of the 95% CI.

**Table 1:** Socioeconomic and demographic characteristics of people who consumed unconventional food plants, edible mushrooms, wild meat, and those not consuming biodiverse foods.

Total sample n = 46,167	Food groups							
	Biodiverse food consumption						Not consuming biodiverse foods (n = 45,439)	
	Unconventional food plants (n = 618)		Edible mushrooms (n = 24)		Wild meat (n = 86)			
	%	95% CI	%	95% CI	%	95% CI	%	95% CI
Gender								
Male	38.76	33.93–43.59	30.57	9.5–51.64	72.87	56.63–89.11	47.98	47.48–48.49
Females	61.24	56.41–66.07	69.43	48.35–90.51	27.13	10.89–43.37	52.02	51.51–52.52
Brazilian region								
North	15.10	10.42–19.78	0	0	55.54	23.30–87.78	8.24	7.76–8.72
Northeast	40.04	32.68–47.41	0.03	0.03–0.09	17.44	01.25–33.64	27.35	26.60–28.10
South	1.19	0.01–2.51	34.06	0.04–67.75	0	–	14.84	14.25–15.44
Southeast	25.28	18.15–32.40	44.85	5.15–84.54	1.47	1.50–4.42	43.58	42.59–44.56
Central	18.39	11.80–24.97	20.81	9.91–51.53	25.55	12.78–63.88	5.99	5.64–6.33
Degree of urbanization								
Urban	72.27	65.79–78,75	100	–	40.76	9.03–72.49	85.68	85.05–86.31
Rural	27.73	21.25–34.21	0	–	59.24	27.51–90.97	14.32	13.69–14.95
Ethnicity								
White	29.29	22.92–35.62	69.65	26.31–100	8.22	1.31–15.13	43.28	42.21–44.31
Black	11.78	07.86–15.65	0	–	9.71	0.94–18.48	10.83	10.19–11.44
Asian	0.16	0.13–0.40	0	–	0	–	0.70	0.49–0.88
Multiethnic	58.64	51.94–65.30	30.35	12.99–73.69	80.09	66.78–93.41	44.74	43.78–45.67
Indigenous	0.13	0.07–0.23	0	–	1.98	0.69–4.65	0.45	0.29–0.58

	Mean	95% CI	Mean	95% CI	Mean	95% CI	Mean	95% CI
Serving size (g)	50.56	41.27–59.84	14.45	8.73–20.26	65.59	57.70–73.48	–	–
Age (years)	42.02	39.59–44.45	47.72	29.40–66.03	34.54	30.25–38.83	39.89	39.55–40.23
Education (years)	8.27	7.68–8.86	14.15	12.95–15.34	6.12	4.39–7.86	8.99	8.91–9.09
Income per capita (USD)^a^	493.56	407.44–579.69	2404.50	1172.01–3,636.77	246.84	114.12–379.57	565.87	541.27–590.48

95% CI = confidence interval of 95%

^a^Quotation to the mean of research period (December 2017).

We observed two different patterns of socioeconomic conditions for people who consumed mushroom and for people who consumed wild meat. Among people who consumed mushroom, mostly were white women living in the South and Southeast, 100% in urban areas, with higher education (average of 14 years of formal education), average per capita income of USD 2404.50, and more than 80% in a state of food security. In contrast, among people who consumed wild meat, mostly were men, black and indigenous people, living in the North and Northeast in Brazil and in rural areas, with an average per capita income of USD 246.84. The sample who related consumption of wild meat had a similar proportion of FS and severe FI, and the age was, in the mean, 13 years less than those who consumed edible mushrooms.

### Predictors of the consumption of unconventional food plants

**Figure 3:**
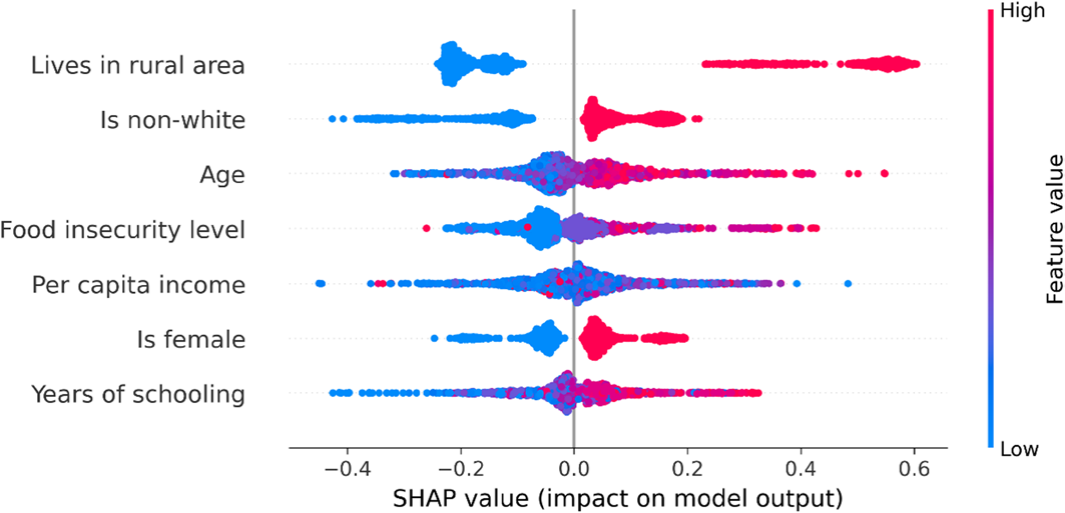
SHAP values for the predictors of unconventional food plant consumption, considering the direction (values to the right of the central axis have a positive impact on consumption) and magnitude (indicated by the colors) of the relationships between the variables.

In the model presented in Fig. 3, living in rural areas, being non-white, being older, living in a household with food insecurity, being a woman, and having more years of schooling are directly related to the presence of UFP in the diet. Comparing who consumed and who did not consume, living in rural areas is the main predictor for consuming UFP while years of schooling is the least influential variable. The values to the right of the central axis have a positive impact on consumption, while the color indicates the magnitude of the impact of the independent variables. In this analysis, it is not possible to determine whether the relationship between income and consumption of UFP was positive or negative.

Supplementing the SHAP analysis, which evaluated the probability of consuming or not consuming UFP, the ZIP model allowed us to evaluate the variables associated with higher or lower consumption of UFP among those who consume them. We observed that, among people who consume UFP, the average of servings was 1.6 daily (range 0–16/day) with significant differences observed for income and area (Table 2), regardless of ethnicity, age, food insecurity, sex, and educational level. In other words, individuals who live in rural areas consumed more servings of UFP per day than individuals who live in urban areas (2 servings/day against 1.5 servings/day) and less wealthy individuals consumed more servings of UFP per day than more wealthy individuals (1.6 servings/day against 1.5 servings/day).

**Table 2:** Effect of socioeconomic and demographic variables on unconventional food plant consumption (UFP).

Covariates	UFP intake-serving/day (Poisson part)		
	Estimation	SE	P-value
Area (rural)	0.43	0.21	**0.042**
Ethnicity (non-white)	− 0.44	0.24	0.066
Age	0.01	0.01	0.140
Food insecurity	0.06	0.10	0.560
Income	− 0.21	0.09	**0.015**
Sex	0.18	0.16	0.257
Educational level	− 0.01	0.02	0.614

Significant values are in bold.

## Discussion

For the first time, we estimated the biodiversity of the Brazilian diet, including its taxonomy, and measured the magnitude of biodiverse food consumption in the Brazilian diet on a national scale. In our sample, the frequency of UFP consumption were higher among women, non-white people, people living in rural areas, and people with income below the national average, in other words, people living in conditions associated with social vulnerability. We also noticed that people living in rural areas and people with lower income consumed more servings of unconventional food plants. However, while it is possible to identify in our sample that the poor and women residing in rural areas use more UFP, it is important to note that this may not represent a significant proportion of the total population in Brazil. Besides that, data on mushroom and wild meat consumption in Brazil is still vague and does not allow us to test hypotheses or even identify consumption predictors. Nevertheless, it seems to show a relationship with well-defined socioeconomic and demographic variables.

### Neglected and invisible food potential

The food biodiversity that was assessed by the NDS-HBS neglected consumption that is invisible, in part, due to the methodological limitations of food consumption research. In the case of wild meat, for example, there are legal implications (Federal Law 9605/98—Environmental Crimes Law), including penalties for people who use native or migratory wild animals without the permission of competent authorities, even for food consumption. However, this legislation is widely recognized as unable to prevent consumption^28–30^, so that in much of the country, the consumption of wild meat is done secretly. In this context, many users do not admit to using or selling products derived from wildlife, as they are aware that this involves illegal activity, an aspect that presents additional difficulties in recording the consumption of these animals.

Another methodological limitation for capturing food biodiversity is the use of the 24-h recall (R24h) as the sole tool for collecting food consumption information. As UFP, edible mushrooms, and wild meat are episodically consumed, complementary methods of consumption assessment would be necessary to analyze their habitual consumption^31^. Many of the species of UFP, edible mushrooms, and wild meat are cultivated or gathered and consumed by traditional Brazilian peoples and communities. The consumption of native mushrooms, for example, is characteristic of the Yanomami people, in a diversity of species that is not, by any means, reflected in the general population^32^. The consumption of wild animals occurs in all regions of Brazil, encompassing a wide diversity of wild vertebrates, being consumed in both rural and urban areas, and by traditional and non-traditional communities^33–38^. In addition, the species of wild animals consumed by these communities vary seasonally^39,40^. Therefore, despite the R24h being applied on non-consecutive days, this tool is not able to capture the influence of seasonal variation on the consumption patterns in different communities.

### The explanatory power of socioeconomic variables

The record of consumption of only seven species of wild animals in our study indicates that animal consumption is underestimated in the NDS-HBS, when considering other studies conducted in Brazil. For example, reviews show that at least 52 species of wild mammals are consumed in Brazil^41^, 39 species of wild birds in the state of Ceará, and 13 species of reptiles in Brazil^34,42,43^. The lack of more comprehensive information on the consumption of wild animals also raises sanitary concerns, as this is one of the main means of transmission of zoonoses^44^.

The food potential of mushrooms is neglected by the Brazilian population, being restricted to a small and particular group. Researchers estimate the existence of about 2189 species of edible mushrooms worldwide, of which 2006 can be safely consumed, and another 183 species that need some pre-processing to make them suitable for consumption^45^. However, we were unable to identify the species consumed in Brazil from the available data, presenting additional challenges in identification compared to wild meat and UFP. Boin and Nunes^46^ identified the influence of socioeconomic factors on mushroom consumption behavior in a sample of 925 individuals living in Portugal. Among the factors, family size, educational level, and gender were the most influential on consumption, corroborating the characteristics we described of the Brazilian population consuming mushrooms. In addition, limited use may be a result of a mycophobic culture. Cardoso et al.^47^ analyzed the diet of Japanese and Japanese descendants living in São Paulo, Brazil, which has the highest mushroom consumption in the country. They discovered that mushroom consumption was unusual, as expected, being higher among the Japanese group compared to the Japanese descendants. Ethnomycological studies show that most indigenous peoples in Brazil are not mycophobic, like other indigenous communities in Latin America^48^. Therefore, we believe that considering the consumption of mushrooms by traditional communities can be strategic to expand knowledge about native Brazilian fungi and promote their responsible use. Researchers worldwide have been striving to include native and cultivated mushrooms in sustainability policies due to their potential to contribute to a sustainable food production system (circular food chains)^49^, reduce food insecurity^50^, and conserve the biodiversity of the planet^51^. If we consider legislation or guidelines for the cultivation, commercialization, and consumption of mushrooms express part of the culture of a country^52^ we can infer that factors such as (1) the absence of a national list of edible native mushrooms in Brazil and (2) the lack of knowledge about the food properties of native and cultivated mushrooms in Brazil may indicate a general resistance to fungi as food in Brazil.

Our analysis shows that living in a rural area, being female, not being white, and having an income below the national average are important predictors of UFP consumption. At the national level, people who consumed UFP seem to be a vulnerable population. The socioeconomic profile of people who consumed UFP was similar to that of individuals who are vulnerable to food insecurity in Brazil, as indicated by a recent national survey^53^, which showed that women, black people, and rural residents are more likely to experience food insecurity. Studies indicate that in rural communities, income is related to the consumption of wild edible plants^14^. Low-income families consume more UFP due to the difficulty in accessing markets and participating in the market economy, as indicated by the study of Reyes-García et al.^54^. In the Asia–Pacific region, UFPs are strongly associated with providing essential micronutrients to individuals experiencing food insecurity, making them a strategic resource in emergency situations, such as climate-related disasters, interrupted crop production, and other forms of food deprivation^55^. In a study^56^ conducted in Kenya, the use of wild edible plants, was tested as a means of enhancing food security for women and children. The study found that consuming wild plants significantly reduced the cost of diets and addressed nutrient gaps throughout the year.

Although, as a cross-sectional study, we cannot confirm the order of the factors, we have a strong hypothesis that the consumption of these plants occurs in contexts of resource use to cope with food insecurity, such as in the case of emergency or famine foods^14,57,58^. Both in and outside of contexts of food insecurity, the consumption of these plants can be observed among populations living in rural areas, largely due to their easy accessibility. According to Cooper et al.^59^, geographical variables such as low population density and high rates of forests and natural areas in Africa, play a more significant role in the collection of wild foods than family income levels or food security status. Similarly, ethnicity is also a predictor of UFP consumption, as these plants are part of the culture of indigenous and traditional communities around the world. In addition to their food importance, UFPs also hold social and cultural value for these communities. Different ethnic groups may have varying levels of knowledge about these plants, as evidenced by a study^60^ in southeast Nigeria that analyzed people's knowledge about plants in relation to socioeconomic factors, including ethnicity. Finally, women are often associated with greater knowledge and consumption of these plants. While there is no consensus on the relationship between gender and UFP consumption, some studies suggest that women are the ones who most use these plants in households, as they are often responsible for family meals. Women's contributions in this regard are significant for the food security of their families and communities^61–63^.

### Possible ways to highlight and promote food biodiversity

Based on our results, we highlight three important strategies to promote the proper use and consumption of food biodiversity in different groups. First, in the context of vulnerable populations, encouraging the commercialization of biodiversity products for income generation is a way to alleviate poverty. Biodiversity product value chains have become increasingly relevant for the conservation and subsistence of local communities. A study^64^ in the Brazilian Northeast analyzed the value chains of two important food biodiversity products, Pequi (*Caryocar coriaceum Wittm*.) and Fava d’anta (*Dimorphandra gardneriana Tul*.), and found that the collection of these products was the main source of income for extractivists,. However, it is important to note that the trade of these products is complex and dynamic, influenced by market relations, political environment, actors involved, and the natural resource in question^65^. Therefore, generalizations are not appropriate without considering the specific context studied. In addition, government support is essential to preserve value chains, through the provision of institutional, technical, and financial support. Second, mobilizing the sustainable use of food biodiversity is essential to make its consumption more appropriate and conscious. Biodiversity for food is indispensable for food security and sustainable development. The FAO report on *the State of the World's Biodiversity for Food and Agriculture* reviewed food and agricultural biodiversity information from 91 countries and identified the promotion of sustainable use of key components of biodiversity as a top priority. This includes supporting sustainable production and management practices, as well as promoting conscious consumption of biodiversity products to increase their demand in the market. However, it is crucial to emphasize the importance of improving the collection of data on the use of these foods at the national level, which constitutes the third strategy that we propose. Although there are no definitive global data, there are estimates available for specific locations, regions, or types of underutilized foods, but it is still difficult to quantify the global contributions of biodiverse foods to diets^66^. Therefore, we recommend increasing investment in ethnobiological studies that evaluate the effects of consuming these foods on human and environmental health, as well as their implications for culture. We believe that promoting conscious and appropriate consumption is a viable approach to promote food biodiversity through national food guidelines based on food.

Besides, we outline a set of possible recommendations to increase sensitivity in capturing biodiversity in the diet and the representation of groups in population surveys. First, to increase the sensitivity of the methods to estimate food consumption, it is necessary (a) include strategic edible resources for food security and biodiversity conservation in lists for food consumption assessment, also considering dietary biodiversity indicators, and (b) investigate consumption assessment in diverse sub-samples of difficult access, such as indigenous peoples, traditional communities, and semi-urban areas, where the trade of wild animals for human consumption is common. Second, for sustainable food consumption promotion, we recommend (c) propagate the knowledge of the nutritional potential of local biodiversity of fauna, flora, funga, and algae with a focus on sustainable development, particularly in the context of food programs and policies; (d) strengthen public policies on food and nutrition security in Brazil to protect indigenous peoples and traditional communities that rely on hunting and collecting natural resources for food uses; and (e) support educational campaigns and resources to increase awareness and understanding of the importance of biodiverse foods for both human health and the environment. Overcoming these limitations can contribute to the improvement of epidemiological and nutritional surveillance systems and promote food security and sustainable food.

## Conclusion

Our findings of low utilization of biodiversity suggest an important mismatch between the rich biodiversity of the country and its representation in the human diet. The biodiverse consumption varies in according to socioeconomic context, as in the case of UFP in Brazil, where the consumption of unconventional food plants may be an indicator of social vulnerability. So, it is essential to consider socioeconomic profiles when developing campaigns to promote the consumption of these food resources, as this can help avoid reinforcing stigmas and perceptions of privilege surrounding food.

## Supplementary Information

Supplementary Table 1.

Supplementary Table 2.

Supplementary Table 3.

## Data Availability

The datasets generated during and/or analysed during the current study are available in the IBGE repository (https://www.ibge.gov.br/estatisticas/sociais/saude/24786-pesquisa-de-orcamentos-familiares-2.html?=&t=microdados). The complete dataset and source code essential for reproducing the results of our machine learning experiments can be readily accessed via the following public repository: (https://github.com/eliasjacob/paper_biodiversity_overlooked).
