# Author Correction: Biodiversity is overlooked in the diets of different social groups in Brazil

**DOI:** 10.1038/s41598-023-36394-9

**Published:** 2023-06-07

**Authors:** Sávio Marcelino Gomes, Viviany Moura Chaves, Aline Martins de Carvalho, Elenilma Barros da Silva, Elias Jacob de Menezes Neto, Gabriela de Farias Moura, Leonardo da Silva Chaves, Rômulo Romeu Nóbrega Alves, Ulysses Paulino de Albuquerque, Fillipe de Oliveira Pereira, Michelle Cristine Medeiros Jacob

**Affiliations:** 1 Department of Nutrition, Federal University of Paraiba, Street Tabelião Stanislau Eloy, s/n, Castelo Branco, João Pessoa, PB 58050-585 Brazil; 2 Multicampi School of Medical Sciences, Federal University of Rio Grande do Norte, Caicó, RN Brazil; 3 Department of Nutrition, School of Public Health, University of São Paulo, Av. Dr Arnaldo, 715, São Paulo, SP 01246-904 Brazil; 4 Restaurante Universitário–Federal University of Para, Rua Algusto Corrêa, 01, Belém, Pará 66075-110 Brazil; 5 Metropole Digital Institute, Federal University of Rio Grande do Norte, Natal, RN Brazil; 6 LabNutrir, Nutrition Department, Federal University of Rio Grande do Norte, Av. Senador Salgado Filho, s/n, Lagoa Nova, Natal, RN 59078-970 Brazil; 7 Escola de Educação e Humanidades, Universidade Católica de Pernambuco, Rua do Príncipe, n. 526, Boa Vista, Recife, Pernambuco Brazil; 8 Museu de Arqueologia e Ciências Naturais da Universidade Católica de Pernambuco, Rua Oliveira Lima, 824, Boa Vista, Recife, Pernambuco Brazil; 9 Departamento de Biologia, Universidade Estadual da Paraíba, Campina Grande, Paraíba 58019-753 Brazil; 10 Laboratório de Ecologia e Evolução de Sistemas Socioecológicos, Departamento de Botânica, Universidade Federal de Pernambuco, Recife, Pernambuco 50670-901 Brazil; 11 FUNGI Research Group, Academic Unit of Health, Education and Health Center, Federal University of Campina Grande, Sítio Olho D’agua da Bica, s/n, Cuité, PB 58175-000 Brazil

Correction to: *Scientific Reports* 10.1038/s41598-023-34543-8, published online 09 May 2023

The original version of this Article contained errors.

In the Methods section, under the subheading ‘Socioeconomic and demographic variables’,

“The latter category (i.e., non-white) included black, asian, multiethnic, and indigenous groups to create a group that potentially combines similar experiences of oppression based on race, as described in Wood et al.^19^ (source: https://doi.org/10.1017/S0023879100009377) and Skidmore^20^ (source: https://doi.org/10.1017/S0022216X00004703).”

now reads:

“The latter category (i.e., non-white) included black, asian, multiethnic, and indigenous groups to create a group that potentially combines similar experiences of oppression based on race, as described in Wood et al.^19^ and Skidmore^20^.”

In addition, the Funding section in the original version of this Article was incomplete.

“This research was supported by the High-Performance Computing Center at UFRN (NPAD/UFRN). Additionally, this study was funded by the CNPq through a research grant to MCMJ (402334/2021-3) and a research productivity scholarship also awarded to MCMJ (306755/2021-1), EJDMN (302668/2020-9), UPA (305285/2020-3), and RRNA (307011/2022-4). AMC is supported by ‘Fundação de Amparo à Pesquisa do Estado de São Paulo’–FAPESP, Brazil (grant #2022/03091-6). The funders played no role in the study design, data collection and analysis, or the decision to publish or pre-pare the manuscript.”

now reads:

“This research was supported by the High-Performance Computing Center at UFRN (NPAD/UFRN). Additionally, this study was funded by the CNPq through a research grant to MCMJ (402334/2021-3) and a research productivity scholarship also awarded to MCMJ (306755/2021-1), EJDMN (302668/2020-9), UPA (305285/2020-3), and RRNA (307011/2022-4). AMC is supported by ‘Fundação de Amparo à Pesquisa do Estado de São Paulo’–FAPESP, Brazil (grant #2022/03091-6). This study was financed in part by the Coordenação de Aperfeiçoamento de Pessoal de Nível Superior - Brasil (CAPES) - Finance Code 001. The funders played no role in the study design, data collection and analysis, or the decision to publish or prepare the manuscript.”

The original Article has been corrected.

