# Supplementary material for: Biodiversity is overlooked in the diets of different social groups in Brazil
Source: Sci Rep. 2023 May 9;13:7509. doi: 10.1038/s41598-023-34543-8 (PMC10170146; doi:10.1038/s41598-023-34543-8)
Supplement: Supplementary file 1 — Supplementary Table 1. [file 41598_2023_34543_MOESM1_ESM.docx]

**Biodiversity is Overlooked in the Diets of Different Social Groups in Brazil**

Sávio Marcelino Gomes, Viviany Moura Chaves, Aline Martins de Carvalho, Elenilma Barros da Silva, Elias Jacob de Menezes Neto, Gabriela de Farias Moura, Leonardo da Silva Chaves, Rômulo Romeu Nóbrega Alves, Ulysses Paulino de Albuquerque, Fillipe de Oliveira Pereira, Michelle Cristine Medeiros Jacob.

**Supplementary Table 1.** List of architectures tested in modeling the evaluated phenomenon.

| model | F1 | BACC | ACC | MCC |
| --- | --- | --- | --- | --- |
| Logistic regression + Catboost | 0,539405 | 0,632686 | 0,659547 | 0,274607 |
| Catboost | 0,49667 | 0,618393 | 0,657605 | 0,258281 |
| Multi-layer perceptron | 0,480307 | 0,608954 | 0,649838 | 0,239408 |
| Support vector machine | 0,550273 | 0,617314 | 0,626537 | 0,232146 |
| Linear support vector machine | 0,555301 | 0,617044 | 0,622654 | 0,230583 |
| Logistic regression | 0,55345 | 0,614078 | 0,61877 | 0,224509 |
| XGBoost | 0,506897 | 0,604099 | 0,629773 | 0,213728 |
| Random forest | 0,479927 | 0,596818 | 0,631068 | 0,205221 |
| Light gradient boosting machine | 0,528951 | 0,599784 | 0,610356 | 0,197643 |
| K nearest neighbours | 0,449378 | 0,567152 | 0,598706 | 0,140056 |
| Decision tree | 0,483254 | 0,565534 | 0,580583 | 0,130473 |
| Stochastic gradient descent | 0,502595 | 0,562837 | 0,565696 | 0,123313 |
