# Supplementary material for: Biodiversity is overlooked in the diets of different social groups in Brazil
Source: Sci Rep. 2023 May 9;13:7509. doi: 10.1038/s41598-023-34543-8 (PMC10170146; doi:10.1038/s41598-023-34543-8)
Supplement: Supplementary file 2 — Supplementary Table 2. [file 41598_2023_34543_MOESM2_ESM.docx]

**Biodiversity is Overlooked in the Diets of Different Social Groups in Brazil**

Sávio Marcelino Gomes, Viviany Moura Chaves, Aline Martins de Carvalho, Elenilma Barros da Silva, Elias Jacob de Menezes Neto, Gabriela de Farias Moura, Leonardo da Silva Chaves, Rômulo Romeu Nóbrega Alves, Ulysses Paulino de Albuquerque, Fillipe de Oliveira Pereira, Michelle Cristine Medeiros Jacob.

**Supplementary Table 2,** Biodiverse foods consumed by the Brazilian population according to the National Dietary Survey - Household Budget Survey (NDS-HBS), 2017-2018.

| **Category** | **#** | **Taxonomic cue** | **Vernacular name** | **Origin** | **Code in the NDS-HBS** | **Provinces where consumption occurs (frequency)** | **Frequency of consumption** |
| --- | --- | --- | --- | --- | --- | --- | --- |
| Unconventional Food Plants | 1 | *Amaranthus viridis* L. | Caruru | Exotic | 6701901 | BA (7), SE (3), PA (1), MG(4) | 15 |
|  | 2 | *Annona* spp. | Araticum | Native | 6805101 | MG (1) | 1 |
|  | 3 | *Artocarpus heterophyllus* Lam. | Jaca | Exotic | 6804101 | BA (28), AL (19), PE (18), MA (12), PB (8), MT (7), MG (5), MS (3), ES (3), RJ (3), PR (2), SP (2), RN (2), PI (2), AC (1), PA (1), CE (1), DF (1), SE (13) | 131 |
|  | 4 | *Astrocaryum aculeatum* G. Mey. | Tucumã | Native | 6601108 | AM (87), PA (14), RO (4), PI (1) | 106 |
|  | 5 | *Attalea speciosa* Mart. | Coco babacu | Native | 6601002 | MA (1) | 1 |
|  | 6 | *Butia* spp. | Butia | Native | 6600912 | PR (1) | 1 |
|  | 7 | *Byrsonima* spp. | Murici | Native | 6807001 | BA (4), PA (3), MA (2), MT (2), AP (1), GO (1) | 13 |
|  | 8 | *Cajanus cajan* (L.) Mill. | Andu | Exotic | 6302602 | BA (21), MG (9), PE (8), TO (5), DF (4) | 47 |
|  | 9 | *Caryocar* spp. | Arroz com frango e pequi | Native | 8579024 | MT (7), TO (4), DF (2), PA (1), MA (1), CE (1), MG (1), SP (1), RS (1), GO (1) | 20 |
|  | 10 | *Caryocar* spp. | Arroz com pequi | Native | 8579033 | MG (11), GO (4), MA (3), PI (1) | 19 |
|  | 11 | *Caryocar* spp. | Frango ou galinha com pequi | Native | 8579149 | GO (25), MT (7), MA (4), MG (3), DF (2) | 41 |
|  | 12 | *Caryocar* spp. | Galinhada com pequi | Native | 8506303 | GO (7), MT (2), TO (1) | 10 |
|  | 13 | *Caryocar* spp. | Pequi | Native | 6806701 | GO (59), MT (14), MG (14), MA (9), PA (8), TO (7), CE (6), DF (6), PI (4), BA (4), MS (3), SC (1) | 135 |
|  | 14 | *Cucurbita* spp. | Semente de abóbora | Native | 6303201 | BA (1), MG (1) | 2 |
|  | 15 | *Genipa americana* L. | Jenipapo | Native | 6804001 | BA (1) | 1 |
|  | 16 | *Hancornia speciosa* Gomes | Mangaba | Native | 6805601 | GO (2), RN (1), MT (1) | 4 |
|  | 17 | *Inga* spp. | Inga | Native | 6806601 | AC (2), AM (2), PA (2), SC (2), GO (1) | 9 |
|  | 18 | *Mauritia flexuosa* L. | Buriti | Native | 6602301 | MA (9), AM (8), AC (3), PA (2) | 22 |
|  | 19 | *Pereskia* spp. | Ora-pro-nóbis | Native | 6710701 | MG (9) | 9 |
|  | 20 | *Psidium* spp. | Araçá | Native | 6805701 | MG (2), BA (1) | 3 |
|  | 21 | *Solanum* spp. | Jurubeba | Native | 6808201 | GO (9), MG (4), ES (3), MS (3) | 19 |
|  | 22 | *Spondias purpurea* L. | Ceriguela / seriguela / siriguela | Exotic | 6806902 | MA (7), PI (2), PE (2), SE (2), BA (2), GO (2), CE (1), PB (1), MG (1), RJ (1) | 21 |
|  | 23 | *Syagrus oleracea* (Mart.) Becc | Guariroba (palmito in natura) | Native | 6704409 | GO (23) | 23 |
|  | 24 | *Syagrus* spp. | Licuri | Native | 6600913 | SE (3), DF (1) | 4 |
|  | 25 | *Syzygium* *cumini* (L.) Skeels | Jamelão (jamburão) | Exotic | 6810201 | BA (2) | 2 |
|  | 26 | *Syzygium* spp. | Jambo | Exotic | 6805301 | AC (5), PA (4), PE (3), ES (3), BA (2), AM (1), RA (1), MA (1), MG (1), RJ (1), GO (1), RO (1) | 24 |
|  | 27 | *Talisia* *esculenta* (Cambess.) Radlk. | Pitomba | Native | 6806401 | MA (7), MG (3), AL (2), RN (1) | 13 |
|  | 28 | *Xanthosoma taioba* E.G.Gonç. | Taioba | Native | 6701401 | MG (24), ES (19), RJ (4), SP (3), RO (1), BA (1) | 52 |
| Edible Mushrooms | 1 | - | Cogumelo in natura | - | 6705301 | SP(5), RN(2), RJ(2), PR(1), GO(1), DF(1), RS(1) | 13 |
|  | 2 | - | Cogumelo em conserva | - | 7700601 | DF(2), RS(1) | 3 |
|  | 3 | - | Champignon em conserva | - | 7700602 | PR(3), RS(2), GO(2), MS(1) | 8 |
|  | 4 | - | Risoto de funghi | - | 8579247 | PR(2), MG(1) | 3 |
| Wild Meat | 1 | Alligatoridae | Jacaré | Native | 7107611 | AC(4), AM(3), PA(2), PI(7), GO(1) | 17 |
|  | 2 | *Chelonoidis carbonaria* Spix. | Jabuti | Native | 7107622 | AC(3), PA(4), AP(1) | 8 |
|  | 3 | *Crax* spp. | Mutum | Native | 7803707 | MT(3) | 3 |
|  | 4 | *Crypturellus* spp. | Nambu | Native | 7802801 | AC(5) | 5 |
|  | 5 | *Cuniculus paca* L. | Paca | Native | 7107603 | RO(2), AC(16), AM(9), PA(11), AP(4), TO(2), MG(1), MT(3) | 48 |
|  |  | *Cuniculus paca* L. | Paca assada à pururuca | Native | 8579201 | PA(1) | 1 |
|  | 6 | *Dasyprocta* spp. | Cotia | Nativa | 7107617 | AC(6), PA(5), MA(7), PI(1) | 19 |
|  | 7 | *Hydrochoerus hydrochaeris* L. | Capivara | Native | 7107615 | AC(1), PA(2), AP(4), MA(3) | 10 |

**Brazilian Provinces:** Acre – AC; Alagoas – AL; Amapá – AP; Amazonas – AM; Bahia – BA; Ceará – CE; Distrito Federal – DF; Espírito Santo – ES; Goiás – GO; Maranhão – MA; Mato Grosso – MT; Mato Grosso do Sul – MS; Minas Gerais – MG; Pará – PA; Paraíba – PB; Paraná – PR; Pernambuco – PE; Piauí – PI; Rio de Janeiro – RJ; Rio Grande do Norte – RN; Rio Grande do Sul – RS; Rondônia – RO; Roraima – RR; Santa Catarina – SC; São Paulo – SP; Sergipe – SE; Tocantins – TO.
