# Supplementary material for: Biodiversity is overlooked in the diets of different social groups in Brazil
Source: Sci Rep. 2023 May 9;13:7509. doi: 10.1038/s41598-023-34543-8 (PMC10170146; doi:10.1038/s41598-023-34543-8)
Supplement: Supplementary file 3 — Supplementary Table 3. [file 41598_2023_34543_MOESM3_ESM.docx]

**Biodiversity is Overlooked in the Diets of Different Social Groups in Brazil**

Sávio Marcelino Gomes, Viviany Moura Chaves, Aline Martins de Carvalho, Elenilma Barros da Silva, Elias Jacob de Menezes Neto, Gabriela de Farias Moura, Leonardo da Silva Chaves, Rômulo Romeu Nóbrega Alves, Ulysses Paulino de Albuquerque, Fillipe de Oliveira Pereira, Michelle Cristine Medeiros Jacob.

**Supplementary Table 2,** Food plants not classified as non-conventional by the experts, consumed by the Brazilian population according to the National Dietary Survey - Household Budget Survey (NDS-HBS), 2017-2018.

| **Vernacular name** |
| --- |
| 1. Mandioquinha salsa (batata baroa) |
| 1. Graviola / jaca-de-pobre |
| 1. Cajá |
| 1. Gueroba / gueiroba (palmito in natura) |
| 1. Cajá manga |
| 1. Açafrão |
| 1. Pitanga |
| 1. Ciriguela |
| 1. Tamarindo |
| 1. Pinhão |
| 1. Chicória |
| 1. Jabuticaba / jaboticaba |
| 1. Jucara |
| 1. Serralha |
| 1. Umbu |
| 1. Alho |
| 1. Bacuri |
| 1. Vinagreira |
| 1. Sapoti |
| 1. Cajarana |
| 1. Jussara |
| 1. Coco ouricuri |
| 1. Bocaiuva |
| 1. Araçá açu (goiaba) |
| 1. Fisális |
| 1. Cabaça |
| 1. Coco pupunha |
| 1. Bertalha |
| 1. Língua de vaca (verdura) |
| 1. Açaí |
| 1. Maxixe |
| 1. Banana-pacovã |
| 1. Castanha do Pará |
| 1. Cará |
| 1. Cebolinha |
| 1. Pinha (fruta de conde) |
| 1. Acerola |
| 1. Lima |
| 1. Mandioquinha |
| 1. Coentro |
| 1. Cenoura amarela (batata baroa) |
| 1. Figo |
| 1. Fruta pão |
| 1. Amora |
| 1. Carambola |
| 1. Nectarina |
| 1. Banana-roxa |
| 1. Fruta de conde |
| 1. Coco da Bahia |
| 1. Palma |
| 1. Papaia |
| 1. Pepininho (maxixe) |
| 1. Abiu |
| 1. Banana-são-tome |
| 1. Fava em grão |
| 1. Pupunha |
| 1. Bacaba |
| 1. Cuxa |
| 1. Broto de feijão |
| 1. Biribá / beribá |
| 1. Caxi |
| 1. Mastruz |
| 1. Piquia |
| 1. Cariru |
| 1. Jambu |
| 1. Abricó |
| 1. Feijão (preto, mulatinho, roxo, rosinha, etc) |
| 1. Banana (ouro, prata, d'água, etc) |
| 1. Feijão de corda |
| 1. Maçã |
| 1. Tomate |
| 1. Laranja (pêra, seleta, lima, da terra, etc) |
| 1. Batata inglesa |
| 1. Batata doce |
| 1. Mamão |
| 1. Mandioca |
| 1. Manga |
| 1. Couve |
| 1. Macaxeira |
| 1. Melancia |
| 1. Abóbora |
| 1. Banana da terra |
| 1. Cenoura |
| 1. Inhame |
| 1. Feijão verde |
| 1. Quiabo |
| 1. Aipim |
| 1. Uva |
| 1. Abacaxi |
| 1. Goiaba |
| 1. Pêra |
| 1. Brócolis |
| 1. Tangerina |
| 1. Abacate |
| 1. Rúcula |
| 1. Couve flor |
| 1. Mexerica |
| 1. Caqui |
| 1. Ameixa |
| 1. Pêssego |
| 1. Morango |
| 1. Limão (comum, galego, etc) |
| 1. Moranga |
| 1. Pimenta malagueta |
| 1. Jerimum |
| 1. Caju |
| 1. Kiwi |
| 1. Rabanete |
| 1. Espinafre |
| 1. Acelga |
| 1. Mostarda (verdura) |
| 1. Repolho roxo |
| 1. Escarola |
| 1. Cana de açúcar |
| 1. Ata |
| 1. Palmito in natura |
| 1. Ervilha em grão |
| 1. Gergelim |
| 1. Uxi |
| 1. Maracujá |
| 1. Couve chinesa |
| 1. Tanja |
| 1. Lichia |
| 1. Tapereba / taperiba / cajá-mirim / cajá-pequeno |
| 1. Inhame caraquento (cara) |
| 1. Nozes do Pará |
| 1. Sementes de girassol |
| 1. Ervilha em vagem |
| 1. Mangalo |
| 1. Castanaha do Brasil |
| 1. Cebola roxa |
| 1. Cupuaçu |
| 1. Pataua |
| 1. Atemoia |
| 1. Maricota |
| 1. Grapefruit / toranja |
| 1. Beterraba branca (acelga) |
| 1. Salsão (aipo) |
| 1. Alho poró |
| 1. Erva doce |
| 1. Agrião |
| 1. Bredo |
| 1. Arroz branco |
| 1. Arroz (polido, parboilizado, agulha, agulhinha, etc) |
| 1. Alface |
| 1. Arroz integral |
| 1. Repolho |
| 1. Beterraba |
| 1. Abobrinha |
| 1. Chuchu |
| 1. Pepino |
| 1. Melão |
| 1. Cebola |
| 1. Jiló |
| 1. Bergamota |
| 1. Almeirão |
| 1. Repolho verde |
| 1. Vagem |
| 1. Berinjela / beringela |
| 1. Amendoim (em gão) (in natura) |
| 1. Lentilha |
| 1. Pimentão |
| 1. Castanha de Caju |
| 1. Linhaça |
| 1. Chia |
| 1. Grão de bico |
| 1. Radite |
| 1. Amêndoa |
| 1. Mimosa |
| 1. Orégano |
| 1. Quinoa |
| 1. Damasco |
| 1. Noz |
| 1. Gengibre |
| 1. Soja em grão |
| 1. Semente de linhaça |
| 1. Cacau (fruta) |
| 1. Cranberry |
| 1. Nabo |
| 1. Trigo em grão |
| 1. Tâmara |
| 1. Groselha |
| 1. Noz pecan |
| 1. Endívia |
| 1. Aspargos fresco |
| 1. Alcaparras |
| 1. Hortela |
| 1. Laranjinha japonesa / quincan / kinkan |
| 1. Cereja |
| 1. Mari |
| 1. Castanha da Índia |
| 1. Castanha portuguesa |
| 1. Avelã |
| 1. Pistache |
| 1. Nirá |
